# AI-Powered Vocalization Analysis in Poultry: Systematic Review of Health, Behavior, and Welfare Monitoring

**DOI:** 10.3390/s25134058

**Published:** 2025-06-29

**Authors:** Venkatraman Manikandan, Suresh Neethirajan

**Affiliations:** 1 Faculty of Computer Science, Dalhousie University, Halifax, NS B3H 4R2, Canada; 2 Faculty of Agriculture, Dalhousie University, Halifax, NS B3H 4R2, Canada

**Keywords:** poultry vocalization, acoustic monitoring, edge AI, TinyML, animal welfare, bioacoustics classification

## Abstract

Artificial intelligence and bioacoustics represent a paradigm shift in non-invasive poultry welfare monitoring through advanced vocalization analysis. This comprehensive systematic review critically examines the transformative evolution from traditional acoustic feature extraction—including Mel-Frequency Cepstral Coefficients (MFCCs), spectral entropy, and spectrograms—to cutting-edge deep learning architectures encompassing Convolutional Neural Networks (CNNs), Long Short-Term Memory (LSTM) networks, attention mechanisms, and groundbreaking self-supervised models such as wav2vec2 and Whisper. The investigation reveals compelling evidence for edge computing deployment via TinyML frameworks, addressing critical scalability challenges in commercial poultry environments characterized by acoustic complexity and computational constraints. Advanced applications spanning emotion recognition, disease detection, and behavioral phenotyping demonstrate unprecedented potential for real-time welfare assessment. Through rigorous bibliometric co-occurrence mapping and thematic clustering analysis, this review exposes persistent methodological bottlenecks: dataset standardization deficits, evaluation protocol inconsistencies, and algorithmic interpretability limitations. Critical knowledge gaps emerge in cross-species domain generalization and contextual acoustic adaptation, demanding urgent research prioritization. The findings underscore explainable AI integration as essential for establishing stakeholder trust and regulatory compliance in automated welfare monitoring systems. This synthesis positions acoustic AI as a cornerstone technology enabling ethical, transparent, and scientifically robust precision livestock farming, bridging computational innovation with biological relevance for sustainable poultry production systems. Future research directions emphasize multi-modal sensor integration, standardized evaluation frameworks, and domain-adaptive models capable of generalizing across diverse poultry breeds, housing conditions, and environmental contexts while maintaining interpretability for practical farm deployment.

## 1. Introduction

The integration of artificial intelligence into monitoring intends to change the landscape of animal welfare, behavioral studies, and environmental control. Of many sensing modalities, acoustic sensing has become a very powerful non-invasive way of analyzing the physiological and emotional states of poultry. When vocalizations are well captured, preprocessed, and analyzed, they can provide biological and behavioral information as digital biomarkers for other indicators, including stress, disease, environmental discomfort, and social–emotional cues [1].

**Figure 1:**
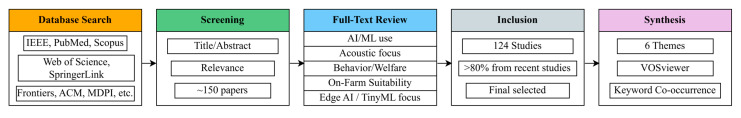
Systematic review pipeline outlining database search, screening, full-text evaluation for on-farm AI acoustic studies, and thematic synthesis from 124 included papers.

This systematic review of literature explores the intersection of bioacoustics, machine learning (ML), and animal welfare, with poultry calls as the contributing data modality. Foundational methods, the particularly relevant ones being Mel-Frequency Cepstral Coefficients (MFCCs) and spectrogram analysis, have set the foundation and have begun to be supplanted with or augmented by methods from deep learning (DL), transfer learning, and self-supervised models such as wav2vec2 and Whisper. This march toward farm deployment is further accelerated by innovations in TinyML, edge computing, and real-time deployment frameworks. Chickens have more than 30 different types of calls [2] that span from distress, mating, predator threats, etc. This makes their vocal repertoires one of the most diverse among domesticated animals. These repertoires may give insights into their emotional and physiological states, thus making vocalization analysis one of the most powerful and non-invasive methods to identify their welfare and state. Vocalizations, from the ethological and communication theory viewpoint, tend to be the selected evolutionary tools for social coordination developed by environmental pressures and flock dynamics. Analyzing poultry vocalizations in that sense aligns with embodied cognition, whereby vocal behavior extends beyond just signaling but becomes a reflection of internal state and context. Several publicly available datasets—such as chick stress vocalizations [3], laying hen audio [4], and raw waveform recordings [5]—have enabled reproducible benchmarking and model comparisons. These and many other datasets are extensively discussed and compared in Section 3, Section 4 and Section 5, alongside the models, feature strategies, and evaluation pipelines they support. This review identifies the trend in methodologies used and key benchmark architectures through a comprehensive thematic synthesis of peer-reviewed studies and identifies critical gaps in current approaches. Increasing importance is put on multi-modal and explainable AI; the dynamic acoustic features rather than static; and standardized datasets and pipelines for reproducibility and generalization. Furthermore, this work adds bibliometric co-occurrence mapping to illustrate evolving thematic structure in the field, thereby aiding in identifying future research trajectories and interdisciplinary collaborations. By bridging computational modeling with ethological relevance, this review aims to inform researchers, practitioners, and technologists about the current state, limitations, and untapped potential of AI-driven poultry vocalization analysis. The review entails a systematic search approach [6] as seen in Figure 1 through IEEE Xplore, PubMed, Scopus, Web of Science, SpringerLink, etc., focusing on research work performed between 2018 and March 2025. The query consisted of various terms related to poultry vocalizations and AI (e.g., “chicken,” “acoustic,” “machine learning,” “CNN,” “Transformer,” “wav2vec”).

**Figure 2:**
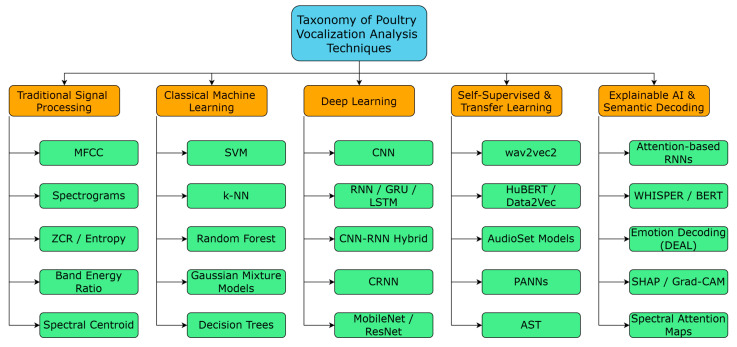
Taxonomy of poultry vocalization analysis methods across five categories, including signal processing, classical ML, deep learning, self-supervised learning, and explainable AI.

In total, approximately 150 papers were examined, of which 124 were deemed relevant for inclusion based on technical rigor and contribution to poultry acoustic sensing. Studies employing ML or signal processing on vocalizations related to welfare, behavior, or disease detection were prioritized and can be referred in the Figure 2. Seminal references on acoustic features and deep learning methods (e.g., MFCCs, attention mechanisms) are retained to establish technical context. The reviewed literature is organized into six main themes: acoustic features, ML/DL models, behavior and stress detection, disease classification, toolkits and pipelines, and on-farm deployment. Notably, over 85% of the references were published between 2020 and 2025, underscoring the rapid growth of this interdisciplinary domain.

## 2. Acoustic Features and Preprocessing Techniques

The meaningful extraction of acoustic features and sound preprocessing techniques are pivotal in animal vocalization analysis. All the reviewed literature indicates that MFCCs, STFT, spectral entropy, and Mel-spectrograms have always been the core components of both traditional and deep learning pipelines. These methods are summarized in Table 1, showing how static features like MFCCs contrast with dynamic representations such as cochleagrams and wav2vec2 in vocalization analysis. The most popular acoustic feature is the MFCC, which has been cited in over half of the papers for the classification of animal sounds. They have been used to characterize vocational sounds from broiler birds, laying hens, chicks, and ducks, and other species, as perceptually relevant frequency information is extracted. For example, Umarani et al. [7], Pereira et al. [8], Jung et al. [9], and Thomas et al. [10] rely heavily on the use of MFCCs for feeding classifiers like LSTM, CNNs, or k-NN for animal sound classification. In a more technical analysis, standard and enhanced MFCC experiments were further elaborated on by Prabakaran and Sriuppili [11] through certain steps of audio signal analysis that included pre-emphasis, windowing, FFT, and DCT; compared multiple MFCC-Hybrid configurations. Davis and Mermelstein [12] compared various speech parameterization methods and concluded that MFCCs outperform others in recognition accuracy for speech signals. This observation favors the continued dominance of the MFCCs in animal sound classification and warrants their use to proceed with poultry vocalization. Contextual cochleagram features proposed by Sattar [13] beat the MFCCs by over 20% in acoustic recognition performance in the presence of environmental noise on the farms, thus raising concerns about the wide acceptance of MFCCs in smart agriculture settings. Puswal and Liang [14] explored the correlation between vocal features and anatomical traits in chickens. However, while different morphological traits between sexes have been noted, the study has discovered a weak correlation between vocal acoustics and physiology, suggesting behavioral factors and context may have a stronger influence on acoustic variability than morphology. This favors the use of dynamic rather than static acoustic features for classification models in poultry.

The input signals for convolutional networks also often employ spectrograms, especially log-Mel spectrograms. The work of Zhong et al. [15], Henri and Mungloo-Dilmohamud [16], Romero-Mujalli et al. [17], Thomas et al. [18], Mao et al. [19], Mangalam et al. [20], Li et al. [21], and Neethirajan [22] analyzed spectrograms for use in CNNs or spectrogram-based embedding studies. STFT parameters cleanly turned high-quality latent space representations with the help of Mel-scaling and z-normalization, particularly as indicated by Thomas et al. [18] and Sainburg et al. [23].

Spectral entropy is gaining ground as a possible indicator or feature for distress. Herborn et al. [24] showed that reduced ratings on the spectral entropy scale of distress calls-from all of which increased calls per day-and long-term welfare and future well-being outcomes in chicks. In the same line, Ginovart-Panisello et al. [25] had fast-induced stress in newly hatched broilers using Butterworth filtered signals and centroid spectral parameters. There are pipelines in a range of past studies to improve preprocessing in real conditions with lots of noise. Tao et al. [26], MFCC, resorted to ZCR and exponential smoothing to filter signals before extracting features. Time masking, SpecSameClassMix, and Gaussian noise augmentation were employed to enhance the theoretical robustness of spectrograms in the works of Bermant et al. [27] and Soster et al. [3]. Comprehensive augmentations like frequency masking and noise injection were incorporated as stated by Mao et al. [19]. Thomas et al. [10] included noise suppression layers into their wider strategy for audio cleaning before deep-mould training.

Besides feature transformation, automated segmentation tools have proven efficient, similar to the benchmark ones in Terasaka et al. [28] and Michaud et al. [4]. Such studies involved comparative works using libraries such as Librosa, BirdNET, or Perch and revealed how BirdNET resulted in a higher F1-score. Merino Recalde [29] developed pykanto, which is a Python library that facilitates semi-automatic segmentation and labeling of large acoustic datasets to use them in deep learning models.

Beyond MFCCs and spectrograms, researchers also seek other acoustic representations. Latent projection techniques were introduced by Sainburg et al. [23], which sidestep traditional hand-crafted features. The importance of embeddings from perusal models trained on raw audio can be illustrated in the work by Swaminathan et al. [30] and Bermant et al. [27]. The representation learned is often superior to the hand-crafted ones. Some studies also use time-domain parameters such as duration, pitch, zero-crossing rate, and energy. For instance, Du et al. [31] extracted nine temporal and spectral features based on source-filter theory to detect thermal discomfort in laying hens. Ginovart-Panisello et al. [32,33,34,35] often included metrics such as spectral centroid, vocalization rate (VocalNum), and variation in spectral bandwidth in examining the environmental impacts and stress in broiler chickens.

**Table 1:** Comparison of static and dynamic acoustic feature sets in animal vocalization studies. Dynamic features such as cochleagram, SincNet, and wav2vec2 exhibit greater robustness in noisy and real-world farm environments, whereas static features like MFCC and Mel-spectrogram perform well in controlled or low-noise settings.

Bermant	Feature Name	Study/Authors	Model Used	Environment	Reported Accuracy	Notes
Dynamic	SincNet	Bravo Sanchez et al. [5]	Raw waveform classifier	Minimal preprocessing	>65% (NIPS4Bplus)	Learns directly from waveform, robust to distortions
Static	MFCC	Umarani et al. [7]	LSTM	General (RAVDESS)	97.22%	LSTM + MFCC for emotion recognition
Static	MFCC	Jung et al. [9]	CNN	General	91.02% (cattle), 75.78% (hens)	Lower for hens—possibly due to background noise
Static	MFCC variants + FFT/DCT	Prabakaran & Sriuppili [11]	MFCC variants	Controlled	94.44%	Comparative setup across MFCC variations
Dynamic	Cochleagram	Sattar [13]	Context-aware classifier	Noisy farm	>20% higher than MFCC	Better adaptability to environmental noise
Static	Mel-Spectrogram	Henri et al. [16]	MobileNetV2	Birdsong (natural)	84.21%	Limited context modeling
Dynamic	Spectral Entropy	Herborn et al. [24]	Entropy analysis	Chick stress study	Qualitative improvement	Captures emotional states during distress
Dynamic	Wav2vec2 Embeddings	Swaminathan et al. [30]	Fine-tuned classifier	Real-world bird data	F1 = 89%	SSL embeddings outperform handcrafted features
Static	MFCC	Bhandekar et al. [36]	SVM	Lab	95.66%	Strong in low-noise environments

Taken together, these publications show that acoustic feature design is still a very lively arena and a pivotal aspect of poultry vocalization analysis. Feature selection can be completely hand-crafted, learned, or hybrid—the chosen approach substantially affects the robustness and generalizability of the model under the field circumstances of relatively noisy, imbalanced, and unlabeled data.

## 3. Deep Learning and Classical Models

A vast majority of studies that have analyzed poultry and animal vocalizations concentrate on supervised classification techniques, which range from traditional machine learning models to the latest deep learning architectures. Depending on the aims of the individual projects, data limitations, and computing setup, MFCCs, spectrograms, or combinations of audio representations are trained in the models.

### 3.1. Classical Machine Learning Models

Some traditional classifiers, such as SVM, RF, k-NN, Naive Bayes, and Gaussian Naive Bayes, have seen their application in the area of poultry sound classification, especially in cases of low data and resource-constrained environments. These applications and their reported performances are summarized in Table 2, highlighting how traditional classifiers continue to play an important role in poultry sound analysis, particularly under low-data or resource-limited conditions. For example, Bhandekar et al. [36] tested four different models (SVM, k-NN, Naive Bayes, and Random Forest) using MFCC features extracted from chicken vocalizations, where SVM scored the best with an accuracy of 95.66%. In another example, Pereira et al. [8] reported 85.61% accuracy with a Random Forest model trained on FFT-extracted features to assess the distress of chicks.

Tao et al. [26] considered SVM, RF, CNN, and k-NN for the recognition of broiler vocalizations using multi-domain features, where k-NN eventually achieved the best result with an accuracy of 94.16% after feature selection. Ginovart-Panisello et al. [37] used Gaussian Naive Bayes in detecting vaccine response classified based on MFCCs and spectral centroid, with an F1-score of 80%. Du et al. [31] applied SVMs to temporal-spectral features toward the detection of thermal discomfort at a sensitivity of 95.1%.

### 3.2. Convolutional Neural Networks (CNNs)

Convolutional Neural Networks (CNNs) have shown tremendous data nowadays in terms of the usage of deep learning architecture for animal vocalization classification. Several studies often apply a standard or customized CNN mechanism to spectrogram inputs for vocal classification. These CNN-based approaches and their reported performances are summarized in Table 3, highlighting both standard and specialized architectures applied to animal vocalization analysis. High performances are realized among birds or poultry via CNNs in vocalization classification by this group of studies, including Zhong et al. [15], Henri and Mungloo-Dilmohamud [16], Romero-Mujalli et al. [17], Mao et al. [19], Mangalam et al. [20], and Ginovart-Panisello et al. [37]. Henri and Mungloo-Dilmohamud [16] compared MobileNetV2, InceptionV3, and ResNet50, with MobileNetV2 achieving 84.21% accuracy. According to Mangalam et al. [20], a lightweight custom CNN (i.e., with ~300k parameters) outperformed fine-tuned VGG16. Mao et al. [19] discovered light-VGG11 with a 92.78% decrease in parameters against reference architectures, which retained 95% accuracy. Further, Ginovart-Panisello et al. [37] used CNNs trained on spectrograms for the detection of stress. In addition, Mangalam et al. [20], Thomas et al. [10], and Mao et al. [19] demonstrate further contributions regarding the value of CNNs with frozen or fine-tuned pretrained backbones.

Some additional specialized applications are as follows:

- Cuan et al. [38,39]: CNN-based detection of Newcastle disease and avian influenza.
- Ginovart-Panisello et al. [25]: CNNs (ResNet) for detection of acute stress based on vocalization and thermographic data.
- Li et al. [21]: ResNet-50 trained on MFCC + Logfbank features for chick sex detection.

### 3.3. Recurrent Models (LSTM, GRU, CRNN)

Research utilizing temporal modeling via RNNs, LSTMs, GRUs, and hybrid CNN-RNN models appears often in the literature dealing with the sequential structure of vocalizations. The models were LSTM and GRU-based, used for species classification and time-series vocal decoding in Umarani et al. [7] and Bermant et al. [27]. Li et al. [21] and Xu and Chang [40] utilized GRUs and CRNNs to classify health conditions and chick sex. Gupta et al. [41] assessed CNN-LSTM, CNN-GRU, and CNN-LMU over large sets of bird vocalizations, with CNN-LMU achieving the best performance. Jung et al. [9] combined CNN with LSTM for vocal classification but reported better performance for 2D ConvNets than for the hybrid model. Huang et al. [42] developed a sequence model to detect poultry feeding behavior based on vocal patterns.

**Table 3:** Performance of deep learning architectures for animal vocalization classification, including CNN, RNN, and attention-based models.

Authors	Model Type	Reported Accuracy
Jung et al. [9]	2D CNN	91.02% (cattle), 75.78% (hens)
Henri et al. [16]	MobileNetV2	84.21%
Romero-Mujalli et al. [17]	DeepSqueak CNN	Detection: 91%, Class: 93%
Mao et al. [19]	Light-VGG11 CNN	95%
Mangalam et al. [20]	Lightweight CNN	92.23%
Hassan et al. [32]	Conv1D + Burn Layer	98.55%
Hu et al. [34]	MFF-ScSEnet (attention)	>96%
Hu et al. [34]	MFF-ScSEnet CNN	>96%
Gupta et al. [41]	CNN-LMU	Best model
Mousse & Laleye [43]	Attention-based RNN	F1-score = 92.75%

### 3.4. Hybrid and Attention-Based Architectures

Emerging trends integrating CNNs with attention mechanisms or various architectural innovations have arisen in recent works. A Conv1D-based classifier with Burn Layers (noise-injection modules) was implemented by Hassan et al. [32] to enhance generalization, leading to an impressive accuracy of 98.55%. Mousse and Laleye [43] established an attention-based RNN for hens’ behavior recognition and reported an F1 score of 92.75%. Huang et al. [33] proposed ASTNet, a spatio-temporal attention network for video saliency detection, which can be adapted for multi-modal poultry monitoring. Hu et al. [34] proposed MFF-ScSEnet, which combines Mel-spectrogram and SincNet features with a squeeze-and-excitation mechanism and more than 96% accuracy over datasets of bird song.

### 3.5. Performance Benchmarks

Several studies conducted model comparisons: Ginovart-Panisello et al. [37] and Thomas et al. [10] have performed both ablation studies and multi-objective training (classification + age estimation). Bermant et al. [27] benchmarked CNNs and RNNs across echolocation and coda recognition tasks and obtained over 99% accuracy. Gupta et al. [41] and Ghani et al. [35] conducted studies to judge the model generalization across species and setups, thereby demonstrating the necessity for a training set that is large and varied. Bianco et al. [44] reviewed ML techniques in acoustics, stressing how, when sufficient labeled data is available, data-driven classifiers like SVMs, Neural Networks, and Gaussian Mixtures outperform traditional signal processing-based techniques, and thus weigh the trade-off between model interpretability and classification accuracy important consideration in their application for acoustic feature selection and hybrid NLP pipelines along with poultry vocal analysis.

## 4. Self-Supervised and Transfer Learning Approaches

As there are not many annotated datasets available in the realm of animal vocalization research, transfer learning and self-supervised learning (SSL) have become the methodologies for successfully improving model generalization, reducing training cost, and improving performance when working under conditions of noise or limited resources. These applications of transfer learning and SSL models in animal vocalization research are summarized in Table 4, illustrating how pretrained architectures enhance performance under data-scarce and noisy conditions. Several studies, mostly focused on poultry and wildlife acoustics, make use of pretrained models, which are commonly developed and fine-tuned for specific species tasks and have been applied to human audio or general bioacoustics.

### 4.1. Transfer Learning with Pretrained CNNs and Audio Embeddings

Studies have utilized transfer learning through pretraining from large-scale datasets like ImageNet or AudioSet before applying the convolutional model to a novel acoustic signal. Some examples include: Henri and Mungloo-Dilmohamud [16], who refined MobileNetV2, ResNet50, and InceptionV3 for bird song classification, with best accuracy (84.21%) corresponding to MobileNetV2. Thomas et al. [10] transferred PANN (Pretrained Audio Neural Network) weights to a multi-objective CNN for broiler vocalization and age estimation. Mangalam et al. [20] compared a custom CNN with fine-tuned VGG16, concluding that the smaller model worked better under field conditions. Li et al. [21] showed that chick sexing tasks conceived from different architectures (ResNet-50, GRU, CRNN), based on breed and feature type, perform variably. McGinn et al. [45] obtained unsupervised feature embeddings derived from the BirdNET CNN to classify within-species vocalizations, emphasizing its strength without retraining. Ginovart-Panisello et al. [37] applied pretrained CNNs to the spectrograms of hens to induce stress response for vaccinated hens.

### 4.2. Transformer Models and Speech Pretraining

Vaswani et al. [46] introduced a completely novel architecture in the form of their Transformer—a new architecture that replaces recurrence with multi-head self-attention to parallelize sequence modeling and capture long-range dependencies in the modeling process. It was developed for language tasks, but later became fundamental for many acoustic modeling frameworks, including wav2vec2 and BERT. Its scalability and efficiency even become more crucial for studies on poultry vocalization that require temporal analyses across different contexts. Admittedly, transformers from natural language processing are quickly finding utility within audio classification tasks. In a more foundational review concerning AI in livestock, Menezes et al. [47] emphasized the increasing role of transformer-based models and large language models (LLMs) such as BERT and wav2vec2 in agricultural applications. Even though the review mainly covered dairy cattle, it highlights the extent to which such architectures could find application in the study of poultry vocalizations, especially in emotion recognition and welfare prediction. Devlin et al. [48] introduced the new language model, a bidirectional Transformer BERT, trained by means of masked language modeling and next-sentence prediction. Just like many language processing tasks, BERT showed astonishing results in several benchmarks, thereby creating the impetus, in automated response systems, for models such as WHISPER and the fine-tuned version of wav2vec2, which are presently being leveraged for poultry vocalization decoding.

Ghani et al. [35] examined transfer learning for large-scale birdsong detection using models like BirdNET and PaSST. The model PaSST, distilled from BirdNET, achieved the highest performance and development in-domain (F1 = 0.704). Swaminathan et al. [30] applied fine-tuning of wav2vec models using bird recordings and a feed-forward classifier against an F1 of 0.89 on C-xeno-canto data. Abzaliev et al. [49] used the trained wav2vec2 (on human speech) to classify dog barks in terms of breed, sex, and context categories, outperforming all-frames models. Sarkar and Magimai.-Doss [50] found speech-pretrained SSL models to perform at par with those trained specifically for bioacoustics, making it feasible to reuse human-centric models. Neethirajan [51] studied OpenAI’s Whisper model for decoding chicken vocalizations to interpret them semantically in terms of token sequences, which were then analyzed by classifiers of sentiment to deduce the emotional states. Morita et al. [52] used Transformer-based models for long-range dependency studies in Bengalese finch songs: eight syllables appeared to be a good context length. Gong et al. [53] introduced the Audio Spectrogram Transformer (AST)—a convolution-free model that uses patch-based spectrogram inputs fed into a Transformer encoder. AST achieved state-of-the-art accuracy across major audio classification benchmarks, thereby emphasizing the potential of attention-based modeling architectures toward structured poultry vocalization analysis.

### 4.3. Self-Supervised Representation Learning

SSL models have made significant inroads into bioacoustic modeling by reducing the dependency on labeled datasets: Baevski et al. [54] presented wav2vec 2.0, which learns by way of contrastive learning and quantization from raw audio latent representations. It serves as the backbone of several follow-up studies, e.g., [30,49]. Wang et al. [55] applied HuBERT segmenting dog vocalizations and performed grammar induction to discover recurring phone sequences that may reveal meaning in sounds of Canine. Mørk et al. [56] tested Data2Vec-denoising, an approach of robust self-supervised pretraining which can yield up to 18% improvements in accuracy over keyword spotting of supervised baselines. Bravo Sanchez et al. [5] employed SincNet, a neural architecture with parameterized sinc filters for classifying bird vocalizations directly from raw audio waveforms. Attaining more than 65% accuracy on the NIPS4Bplus dataset with minimal preprocessing, this research shows the efficacy of raw-signal-based models for the lower complexity of attack-recognizing classification of poultry vocalizations. In personalized adaptive fine-tuning, Brydinskyi et al. [57] indicated that only 10 min of data from an individual could fine-tune wav2vec2 to reduce word error rates: about 3% for natural voices and as much as 10% for synthetic. In personalized adaptive fine-tuning, Brydinskyi et al. [57] indicated that only 10 min of data from an individual could fine-tune wav2vec2 to reduce word error rates: about 3% for natural voices and as much as 10% for synthetic.

**Table 4:** Reported performance of transfer learning, self-supervised learning (SSL), and AutoML strategies in animal and bioacoustic vocalization analysis.

Authors	Model/Strategy	Reported Performance
Bravo Sanchez et al. [5]	SincNet	>65% accuracy
Thomas et al. [10]	PANN + CNN	Balanced Accuracy = 87.9%
Swaminathan et al. [30]	Fine-tuned wav2vec2	F1 = 89%
Ghani et al. [35]	PaSST (Transformer)	F1 = 70.4%
Abzaliev et al. [49]	Pretrained wav2vec2	Outperformed all-frames models
Mørk et al. [56]	Data2Vec SSL	+18% accuracy vs. supervised baseline
Brydinskyi et al. [57]	Personalized wav2vec2	WER decreased ~3% for natural, ~10% for synthetic)
Tosato et al. [58]	AutoKeras NAS (Xception)	Outperformed ResNet, VGG, etc.

Wav2vec2 performs better than many traditional models in poultry call detection because of its combination of contextualized audio embeddings and contrastive self-supervised training. In general, the MFCC pipeline depends on handcrafted features, but wav2vec2 learns deep representations from a raw waveform by predicting masked latent representations. In this way, the model is able to catch subtle temporal patterns and contextual variations in vocalizations and distortions that degrade standard features in a noisy farm environment. Its fine-tuning possibilities with limited labeled data also make this model apt to be used in low-resource domain problems such as poultry welfare monitoring. Similarly, SincNet performs better over several CNN-based methods due to its ability to learn sinc-based filters that are constrained to represent meaningful frequency bands that are valid frequency bands. This inductive bias enables the model to extract frequency-specific features that are physiologically relevant to bird calls while reducing the parameter search space, thus enhancing generalization across small datasets. Lastly, it operates on the raw waveform directly, avoiding any possible errors introduced in transformations to the spectral domain, such as STFT or Mel-scaling, giving the classifier increased resilience to varying acoustic distortions encountered in the real world.

While models like wav2vec2 and Whisper, fine-tuned for poultry vocalizations, perform exceedingly well, one should observe that their original training was always conducted on human-speech corpora. The structure, phoneme inventory, and temporal dynamics of animal sounds are far from those of human speech. Consequently, although such systems can offer a generic resolution to acoustic feature extraction, the semantic alignment and acoustic priors engineered for human speech do not offer the best clues for the decoding of emotional or behavioral cues speciated to poultry. For instance, spectral bandwidth and non-verbal call structures of birds lack phonetic segmentation assumptions that human speech models rely heavily upon. Mismatches like these become sources of acoustic noise on downstream tasks, which limits zero-shot generalization to presence across unseen animal domains.

### 4.4. AutoML and Neural Architecture Search (NAS)

In addition to the manual transfer learning, some studies employ an active nudging from automated approaches in discovering models: Tosato et al. [58] established an optimal Xception architecture for classifying bird vocalizations by using AutoKeras, which is better than MobileNetV2, ResNet50, and VGG16. Gupta et al. [41] presented the results of exploring a number of deep models on the Cornell Bird Challenge dataset, including CNN-LSTM and CNN-LMU, with CNN-LMU achieving the peak accuracy on Red Crossbill calls. The Top performing Classifiers are reported in Table 5 and Table 6 respectively.

These studies in the aggregate validate the power of pretrained and self-supervised models in enabling accurate, efficient, and scalable animal vocal analysis. Such crossroads include vision-based CNN backbones, language-inspired transformers, or SSL-driven embeddings, where cross-model transfer leads to generalizable, low-data animal sound classification, especially important when annotating precision-livestock contexts, since it is often very time-consuming and costly.

## 5. Emotion, Behavior, and Stress Detection

### 5.1. Stress Detection via Acoustic Signatures

Well-established evidence exists for stress-related modifications of vocal parameters. One of the very few earlier spectrographic studies on chicken vocalizations was undertaken by Collias and Joos [59], who correlated call types (distress calls, clucking, roosting) with relevant behavioral contexts. They found that calls given with descending frequency were often interpreted as distress calls, whereas those with ascending contours often indicated that they were more pleasurable. This important early study laid the groundwork for behavioral correlates of acoustic markers used in avian welfare research. In laying hens, acute stress was detected using a combination of thermographic imaging and CNN-based spectrogram classification by van den Heuvel et al. [60]. This revealed a beak and comb temperature reduction and decreased call rate following stressor exposure. In a similar fashion, Ginovart-Panisello et al. [25] showed that prolonged fasting caused an alteration of vocalizations in chicks, with call rate (VocalNum) and spectral centroid and bandwidth being significantly altered in comparison to fed controls.

In testing the validity of spectral entropy, Herborn et al. [24] found strong links between entropy and welfare outcomes in the long term (reduced weight gain and increased mortality). Sound calls of domestic chicks during isolation were studied by Collins et al. [61], who related these to various levels of emotional arousal as represented by loudness, frequency, and duration. Lev-Ron et al. [62] taught an artificial neural network to classify responses in vocalizations from broilers subjected to environmental stressors, including cold, heat, and wind. The model accuracy was further enhanced by incorporating variables such as age and waveform length to achieve a mean average precision (mAP) of 0.97. Thus, this approach can be scaled up for stress detection in poultry welfare. The effects of auditory stimuli—including classical music and mechanical noise—were studied by Zhao et al. [63] on fear responses and learning in laying hen chicks. Moderate-level Mozart music exposure caused reduced fearfulness, whereas exposure to high-intensity sound impaired learning and increased stress. The emotional response of hens to their chicks in distress was studied by Edgar et al. [64], who found an increase in heart rate, alertness, and maternal vocalizations of hens when distress was simulated in their chicks by air puffs. This suggests that hens can sense offspring distress and react accordingly, providing support for emotional contagion and further emphasizing the use of vocal cues for welfare inferences in poultry.

**Table 6:** Top 5 disease and condition-specific detection models.

Model (Author)	Reported Performance	Target Condition	Species	Notes/Strength	Reference
Thermal Discomfort SVM (Du et al.)	95.1% sensitivity	Heat stress	Laying hens	Time-frequency features; simple yet effective	[31]
DPVN CNN (Cuan et al.)	98.5% accuracy	Newcastle Disease	Chickens	Spectrogram-based; high accuracy	[38]
CNN for Avian Influenza (Cuan et al.)	97.5% accuracy	Avian Influenza	Chickens	Frequency filtering + data augmentation	[39]
Whisper Model (Neethirajan)	Token-level emotional decoding	Emotional/physiological states	Laying hens	NLP-based; interprets emotion from calls	[51]
ANN Stress Classifier (Lev-Ron et al.)	mAP = 0.97	Heat, cold, wind stress	Broilers	Age + waveform inputs improved precision	[62]

### 5.2. Behavior and Reward-Related Vocalizations

Behavioral responses are mirrored in voice patterns. Zimmerman [65] first worked on the “gakel-call” in hens and established linkages with the emotion of frustration that stems from blocked behaviors. More recently, Zimmerman and Koene [66] demonstrated that calls in hens vary depending on the reward anticipated (mealworms, food, substrate), where the frequency shifts in the calls associated with food are related to the expected reward’s valence. A human study conducted by McGrath et al. [67] revealed that people could identify the chicken calls reliably associated with rewards, indicating the presence of semantic information encoded within the calls. Neethirajan [68] also studied this topic with the WHISPER model, confirming that token-based patterns in chicken distranquil vocalization correlated to emotion. Abzaliev et al. [69], in their turn, analyzed vocalizations in the Japanese tit (Parus minor), specifically focusing on phoneme structure classification Via machine learning that will indeed allow for the differentiation of different call types. The training based on validation with human-labeled data will be the major assist in commissioning and developing a real-time automatic classification system for structured communication in birds. In this regard, such investigations could facilitate the transfer of similar models for the detection of poultry call types, for which structured elements may encode important behavioral or emotional states. Schober et al. [70] compiled an extensive and rich acoustic repertoire of Pekin duck vocalizations according to varying stimuli, the sex of the subject, and group configurations. This study applied statistical methods, including ANOVA, cluster analysis, and canonical discriminant analysis, yielding the identification of 16 distinct vocal types linked to behavioral and environmental contexts. Results demonstrate that vocal diversity and sex-specific patterns can serve as proxies for indicating behavioral correlates, in parallel with call-type variation within poultry.

### 5.3. Emotion Recognition Models

Emotion decoding has been taking advantage of advanced AI models: Neethirajan [71] reviewed the integration of NLP and sentiment analysis with acoustic sensing for animal emotional detection, proposing hybrid AI systems based on thermographic and vocal inputs. With collaborative annotations by psychologists and veterinarians, Cai et al. [72] developed the DEAL model (Deep Emotional Analysis Learning) to interpret emotional states such as hunger and fear in chickens. Ginovart-Panisello et al. [37] identified post-vaccine anxiety in hens by extracting MFCC and spectral centroid features into a GNB classifier. The classifier obtained an F1-score of 80%, and moreover, experimentally reduced stress during anti-inflammatory treatment. Du et al. [31] reported a strong correlation between thermal distress and squawking/alarm calling in hens (e.g., squawk–THI: R = 0.594), within an SVM setting applied to time-frequency outputs. Gavojdian et al. [73] introduced BovineTalk, a deep-learning explainable ML framework for emotional valence and individuality characterization in dairy cow vocalizations. They reported accuracies of 89.4% for distinguishing high- from low-frequency calls for affective state classification and 72.5% for cow identification using GRU-based models. The methodology has cross-species relevance for poultry emotion recognition, either on interpretable acoustic features or spectrogram-based modeling. Lavner and Pérez-Granados [74] underlined emerging techniques in passive acoustic monitoring (PAM) for emotional state estimation, pointing to foundational models and threshold-free density estimation tools.

### 5.4. Behavioral State and Health Linkages

Not only does behavioral analysis work through sound for emotion, but it also demarcates behavioral activities. With formant structure and pitch-based features, Huang et al. [42] have established a 95% accuracy rate for identifying episodes of eating behavior in chickens. Using attention-based RNNs, Laleye and Mousse [43] classified laying hen behaviors with an F1-score of 92.75%. Fontana et al. [75] found a negative correlation between broiler vocal frequency and weight, thus establishing an association between acoustic cues and physiological growth. Karatsiolis et al. [76] proposed a non-invasive farm monitoring system that uses vocal, visual, and environmental sensor data to interpret Flock-wide psychological states. Manteuffel et al. [77] reviewed how vocal correlates—like call frequency and formant dispersion—indicate both positive and negative emotional states in multiple species of livestock. Güntürkün [78] reviewed the avian nidopallium caudolaterale (NCL), which, functionally similar to mammalian prefrontal cortex, is involved in decision-making, executive control, and behavioral flexibility. Thus, forming a neuroanatomical basis for understanding poultry vocal behavior complexity, particularly when being stressed, in cognitive load, or interest state. Galef and Laland [79] have considered mechanisms of social learning such as imitation and local enhancement across animal species and their contribution to behavioral adaptation and cultural transmission. This provides theoretical justification for researching social influences on vocal behavior in poultry, such as peer-induced stress responses and learned vocal cues. Rugani et al. [80] recorded that 3-day-old chicks possess proto-arithmetic skills, opting for larger object sets during occlusion-based tests. This early cognitive ability suggests that vocal responses in chicks may encode quantitative or perceptual awareness, further legitimizing studies of poultry behavior that model numeracy-linked vocal characteristics.

### 5.5. Vocal Indicators of Mental State and Social Emotion

Emotion detection of poultry Via vocalization can be meaningfully contextualized using established frameworks such as the Five Domains model (nutrition, environment, health, behavior, and mental state) [81]. In particular, vocal measures of distress, anticipation, and contentment correspond to a kind of “Mental State” domain—difficult to quantify objectively, yet accessible for study with machine learning—allowing one to assess emotions without any invasion. These acoustic measures operate to bridge the gap between visible behavior and internal affective states, yielding a more composite view of welfare. From here, we assert that emotional contagion—the affective state of one individual induces a similar affective response in others—has some emergent relevance for poultry welfare studies, with one being that distress calls offered by one chick can raise vocal stress markers in cage mates, indicating a viable emotional space that can be mapped using a group acoustic approach [82]. If such social-affective dynamics could be detected reliably, they may feed into welfare protocols oriented toward interventions at the flock level. Also, convincing evidence emerging from ethology indicates that hens respond differentially to the vocal cues of their chicks, implying maternal empathy. Thus, the possibility exists of quantifying cross-individual emotional synchrony by utilizing acoustic AI to analyze the call-and-response interaction between hens and their chicks. Thus, this opens entirely new avenues for affective computing and animal cognition, stressing the need to now specifically consider how machine learning systems developed for farm animals not only classify individual vocalizations but also discern social and relational emotional cues that seem to become embedded in such vocal interactions.

## 6. Disease Detection and Health Monitoring

Acoustic analysis is a non-invasive alternative to traditional diagnostics for detecting disease, discomfort, and other mostly physiological anomalies in poultry. Many research studies have employed machine learning models to find health-related vocal markers, to assess disease progression, and to validate the effectiveness of intervention strategies.

### 6.1. Disease-Specific Detection via Vocal Cues

Specific pathogen vocalization signatures have been identified in various studies, including Serbessa et al., who reviewed the clinical syndromes, modes of transmission, and control methods for the most common poultry and pig diseases [83]. This would create an excellent foundation for to interpretation of vocal biomarker correlates for specific health statuses, with comparisons made from different species and disease types. Such a baseline would be important in the AI modeling of automated disease detection through vocalization analysis. Cuan et al. [38] proposed a Deep Poultry Vocalisation Network (DPVN) where Newcastle disease was identified with 98.5% accuracy through calls of infected to healthy chickens. In a subsequent study, Cuan et al. [39] trained a CNN (CSCNN) on spectrograms resulting from avian influenza-infected chickens, achieving 97.5% accuracy, with preprocessing including frequency filtering and time-domain augmentation. Xu and Chang also [40] proposed a hybrid model for deep learning fusing vocal and fecal image features for poultry health diagnosis, which gives the highest accuracy compared to single-modal models. Neethirajan [51] used Whisper, which took chicken vocalizations and created token sequences that were sentiment-scored to identify emotional states and physiological states. Adebayo et al. [84] were able to provide a real-world dataset from over 100 chickens for 65 days. Acoustic changes appeared in untreated birds’ calls for 30 days and were often associated with respiratory problems, making it significantly important to establish a baseline for future modeling of disease-related acoustics.

### 6.2. Physiological Monitoring and Comfort Assessment

Health monitoring also includes assessments of thermal comfort and general well-being. Du et al. [31] used spectral features for the prediction of heat stress in hens, which proved to be more than 95% sensitive and could relate the call type to the Temperature-Humidity Index (THI). The study by Li et al. [21] was able to identify chick sex by feature combinations of MFCC, logfbank, and spectrogram across breeds, reporting high accuracy through ResNet-50 and GRU. Puswal and Liang [14] explored the relationship between vocal features and anatomical traits in chickens. The presence of morphological differences based on sex was observable, but it did not display a strong correlation between vocal acoustics and physical traits, indicating behavior and context are likely causes of acoustic variance more than morphology. Thus, it may strengthen dynamic compared to static acoustic features in poultry classification models. He et al. [85] reviewed early detection of diseases by means of sensors and proposed acoustic sensing as one answer that is emerging but underused for monitoring clinical symptoms. Mao et al. [19] made a lightweight convolutional neural network that can monitor in real time the distress of chickens with accuracy above 95% in validation from recordings performed in noisy conditions. Soster et al. [3] trained a CNN built from more than 2000 broiler vocalizations in the detection of four call types, including distress calls, achieving a balanced accuracy of 91.1%. Thomas et al. [10] created a dual-objective CNN to classify calls and estimate broiler age, thus showing that the vocal patterns change with development and may indicate health status. ChickenSense, a piezoelectric audio sensing device married to a VGG16 CNN, has been developed by Amirivojdan et al. [86] to estimate the feed intake. The model predicted intake at 92% accuracy and a margin of error of ±7%, thus supporting a sound proxy for metabolic state.

### 6.3. Real-World Deployment Considerations

Deployability and robustness form important attributes for practical applications. For instance, the implementation of the TinyML model for monitoring chicken health highly effective approach under varied health and environmental conditions, been demonstrated by Srinivasagan et al. [87] at edge devices. Huang et al. [42] linked vocal changes to physiological states such as hunger and satiety using formant and pitch dynamics to detect feeding behavior.

These studies illustrate the viability of using vocalizations as digital biomarkers for disease, thermal stress, respiratory issues, and overall well-being. Combining bioacoustics with embedded AI models and sensor fusion holds strong promise for continuous, non-invasive health monitoring in poultry farms.

## 7. Automated Pipelines and Toolkits

The availability of large-scale open access bioacoustic data has triggered the need for automated pipelines and toolkits to process, annotate, and analyze vocalizations with little manual effort. In this section, systems and frameworks are discussed that fit into the streamlining of data-preprocessing machine-learning pipelines intended for model training and inference in the analysis of animal sounds.

### 7.1. End-to-End Tools for Bioacoustics

**Figure 3:**
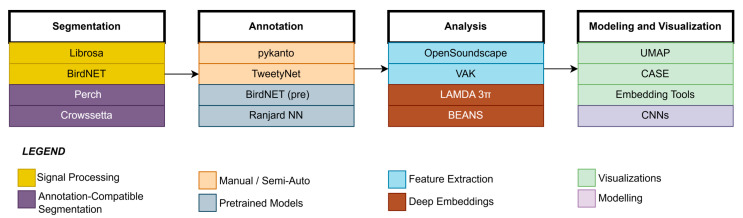
Workflow of Bioacoustic Analysis: Segmentation to Modeling using Specialized Tools.

Bioacoustic software tools for automating large parts of the workflow have recently emerged as we can see in Figure 3. Gibb et al. [88] described a robust overview of passive acoustic monitoring (PAM) pipelines from sensor hardware to acoustic inference. The role of convolutional neural networks (CNNs), unsupervised clustering, hidden Markov models (HMMs), and cross-correlation techniques has been emphasized for scalable ecological assessment. It also addressed challenges like detection uncertainty, model transferability, and the need for standardized datasets for deployment of automated poultry monitoring systems. Schneider et al. [89] presented the clustering and analysis of sound events (CASE), where 48 clustering methods and audio transformations for animal vocalizations were compared. CASE incorporates windowed, multi-feature extraction and serves as the benchmarking tool for unsupervised vocal classification. Thomas et al. [18] describe a practical guide that implements Short-Time Fourier Transform (STFT) and Uniform Manifold Approximation and Projection (UMAP) embeddings to build low-dimensional representations of animal calls and gain insights into mislabeling, clustering quality, and interactive visualization. Merino Recalde [29] has developed pykanto, a Python library for large acoustic dataset management. It contains segmentation, semi-supervised labeling, and deep model integration, thus speeding up reproducibility in the pipeline. Nicholson [90] developed Crowsetta, a Python package that converts several annotation formats (e.g., Praat, Audacity, Raven) into a standardized structure, which is compatible with analysis tools like vak and pandas. This interoperability simplifies vocal dataset processing and enhances reproducibility of the analysis across bioacoustic pipelines; hence, it is very beneficial for studies involving different poultry call types. Lapp et al. [91] developed OpenSoundscape, a Python Toolbox for the detection, classification, and localization of biological sounds, through a synergy of machine-learning principles and signal processing. BirdSet, presented by Rauch et al. [92], is a large dataset consisting of more than 6800 h of avian recordings. In that paper, six deep models were benchmarked, and the source code is available on Hugging Face to promote reproducibility and model evaluation under covariate shift.

### 7.2. Acoustic Segmentation and Dataset Cleaning

For reliable segmentation, high-quality training datasets are essential. In this context, et al. [28] compared four segmentation tools in order, namely, Librosa, BirdNET, Perch, Few-shot Bioacoustic Event Detection, and concluded that BirdNET was the most accurate. Michaud et al. [4] proposed a DBSCAN and BirdNET-based unsupervised classification method, which ultimately filtered label noise from song datasets, thereby enhancing downstream model performance. Sasek et al. [93] introduced a deep supervised source separation (DSSS) framework specialized for site-specific bird vocalization data. A considerable enhancement in separation quality and reduction in downstream labeling errors were achieved by training the ConvTasNet and SuDORMRFNet models using a semi-automated pipeline based on BirdNET, PANNs, and manual filtering. This method shows that integrated pipelines hold great promise when studying poultry calls among other confounding noises in farming settings.

An unsupervised syllable classification approach was developed by Ranjard and Ross [94] with evolving neural networks for the large-scale annotation of bird songs. TweetyNet, a neural network that segments birdsong spectrograms into syllables, was developed by Cohen et al. [95] through end-to-end training, demonstrating good generalizability across species. Lastly, Sethi et al. [96] demonstrated how automated pipelines can scale up biodiversity monitoring by using a BirdNET model pretrained on 152,000+ hours of global audio and manually calibrating detection thresholds for over 100 species.

### 7.3. Specialized Detection Systems

Lostanlen et al. [97] created BirdVoxDetect (BVD), a freely available system for detecting nocturnal flight calls of birds. It harnesses a multitask CNN to extract features for classification, while faults in the sensor are detected using a random forest model. Michez et al. [98] reported a methodological pipeline using UAS for airborne bioacoustic monitoring of birds and bats. It evaluates drone height and motor noise impacts on call detection rates, with a particular focus on ultra-high frequencies. Their protocol offers a standard for airborne data collection in vocalization-based biodiversity and behavior studies, which may even have further applications in poultry farm surveillance. Guerrero et al. [99] created an unsupervised clustering pipeline (LAMDA 3π) designed for ecological soundscapes. Their approach divides the spectrograms and groups species-specific acoustic clusters (sonotypes), which makes biodiversity assessments possible without labeled data. ChickTrack is the system developed using YOLOv5 plus Kalman filtering in real-time chicken tracking, which is integrated with the monitoring of behaviors using over 3800 annotated frames from Neethirajan [100]. Bermant et al. [27] present a hybrid pipeline with CNNs for echolocation click detection and RNNs for time-series analysis of sperm whale vocalizations, where transfer learning on proxy tasks allows achieving high-accuracy downstream classification. Berthet et al. [101] reviewed the application of linguistic theory (syntax, semantics, pragmatics) in animal communication systems and proposed analytical pipelines that include linguistic models into neuroethological data. Hagiwara et al. [102] presented BEANS (Benchmark of Animal Sounds). It is a benchmark that combines 12 different datasets available in public, covering birds, mammals, anurans, and insects, and sets up classification and detection benchmarks in order to promote standardized evaluation in the field.

These toolkits and pipelines will bring a paradigm shift in the field of animal acoustic analysis, away from individualistic task-specific models toward scalable, generalizable frameworks with standardized data, reproducible pipelines, and automated annotation capacities.

## 8. On-Farm Deployment and Edge AI

For real-world applications of acoustic monitoring in poultry and livestock, it is essential that machine learning models operate reliably under field conditions. Such system requirements are to be self-sufficient and robust in handling noise and power-efficient operation with low-power edge devices or embedded hardware. All those facts made a strong reflection of the dominant trend in research toward practical and affordable solutions in smart agriculture.

### 8.1. TinyML and Embedded Inference

With edge AI, mainly through TinyML, real-time inference is performed directly on equipment deployed at farms (Table 7). In this way, Srinivasagan et al. [87] trained their tiny machine learning models for chicken vocalization using these low-power processors, thus managing memory limitations while maintaining accuracy for multiple health status conditions. The ChickenSense system is a fusion of piezoelectric sensors and the VGG16 model, monitoring the feed intake acoustics of chickens with 92% classification accuracy in +/−7% estimation error (Amirivojdan et al. [86]). Using phase-coding and Gaussian classifiers such as SVM and k-NN on hardware of Raspberry Pi, Bhandekar et al. [36] designed a real-time monitoring system for analysis with synchronized video and audio tracking. Huang et al. [42] developed a module of vocal formants to detect the feeding behavior in noisy field conditions.

TinyML frameworks like TensorFlow Lite Micro, Edge Impulse, and Syntiant now allow optimized models, for example, quantized CNNs or shallow Transformers, to be deployed on low-power microcontrollers such as ARM Cortex-M and ESP32 [103]. Models like these achieve the real-time classification of poultry vocalizations, consuming as little energy as 1–10 mW for continuous monitoring without draining battery-operated IoT systems. In contrast, cloud-based pipelines require constant audio streaming and network bandwidth, which not only increases operational costs but also introduces risks of data leakage, latency bottlenecks, and reliance on external connectivity, particularly problematic in rural farm settings [104].A detailed comparison is present in Table 8.From an AI systems perspective, edge-AI deployments promise better autonomy and resilience, primarily when combined with local feedback loops that might alert farmers about abnormal distress calls. Yet, how viable edge solutions become is largely dependent on the trust and interpretability underpinning them from the perspective of the farmers. Transparent models with explainable outputs, such as call-type labeling and emotion tagging, complemented by local visualization dashboards, will boost the acceptance level of farmers, particularly if privacy-preserving inference methods and fail-safe precautions at the device level are in place.

### 8.2. Robustness to Noise and Uncontrolled Environments

Studies that have addressed the effects of noise and changing environments: Mao et al. [19] employed their lightweight CNN (light-VGG11) for time-continuous recordings and real-farm conditions, confirming its robust performance with over 95% accuracy. Mangalam et al. [20] used on-site smartphone recordings in Indian farms, yielding a 92.23% accuracy rate on three vocalizations by using a lightweight CNN. Goyal et al. [105] dealt with a systematic review in smart poultry farms, particularly highlighting computer vision, IoT, and AI’s role in real-time decision support systems and low-cost deployment. Karatsiolis et al. [76] also proposed something similar, where a multi-modal system, vocal and visual environmental sensor models, is designed to perform the assessment of communal flock welfare using a completely non-invasive procedure.

### 8.3. Sound as a Proxy for Behavior and Environment

Long-term field studies conducted by Ginovart-Panisello et al. [106,107,108] have illustrated how vocal features (e.g., peak frequency, MFCCs) correlate to temperature, humidity, CO_2_ levels, and ventilation conditions across different production cycles. Such studies, therefore, prove the feasibility of passive acoustic monitoring for environmental assessment and flock health systems. Ginovart-Panisello et al. [37] showed that acoustic responses to vaccination can be automatically tracked under farm conditions, even in the absence of labeled emotional categories. In response to fasting stressors in commercial hatcheries, Ginovart-Panisello et al. [25] tracked call rates and spectral features in real-time.

Niu et al. [109] reviewed avian visual cognition and associated brain pathways—the entopallium and visual Wulst. Their findings corroborate birds’ advanced object recognition and tracking capabilities, which provide a neural basis to integrate visual and acoustic signals into behavior monitoring systems. Such integration finds utmost importance in smart poultry surveillance platforms

### 8.4. Deployment-Friendly Design Practices

Many studies involve optimization to reduce model size, boost energy efficiency, or simplify their architecture:

- Mao et al. [19] reduced the total number of parameters by 92.78% against the standard VGG11.
- Hassan et al. [32] introduced Burn Layers (noise-injection modules) to improve generalization under deployment noise.
- Ginovart-Panisello et al. [60] combined thermographic imaging with CNN-based vocal classifiers to provide an in-field assessment of acute stress in a non-invasive manner.

**Figure 4:**
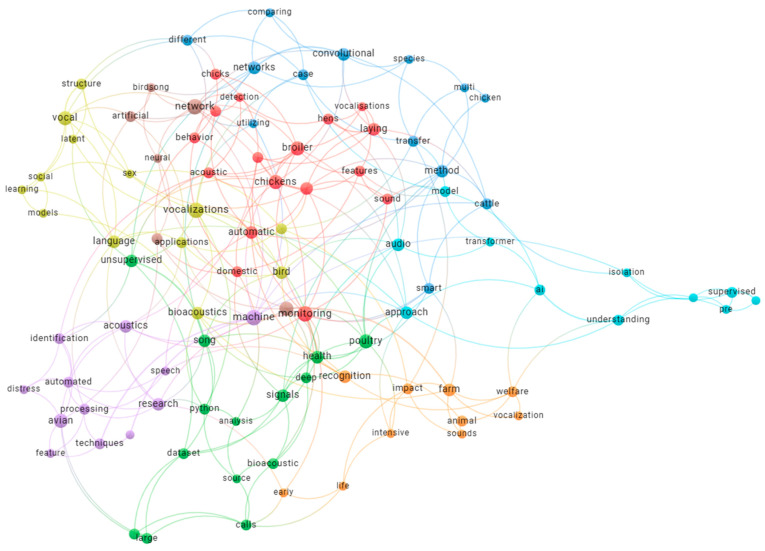
Keyword co-occurrence network showing thematic clusters in livestock vocalization research. Node size indicates keyword frequency, while colors represent distinct research themes such as poultry monitoring, acoustic analysis, and deep learning approaches.

These studies demonstrate that conjoining edge AI with robust and lightweight architectures is not only possible but a necessity for real deployment in commercial poultry production systems. Continuous monitoring under the decision-making process in a non-invasive and interpretable manner, and meeting farm constraints, is fast becoming the norm within smart livestock farming. Finally, a Keyword Cooccurance network Map is shown below in Figure 4.

## 9. Discussions: Challenges, Gaps, and Future Directions

The current situation in the field of Bioacoustics and analysis leaves an important requirement for further research to identify and fine-tune the limitations associated with reproducibility, generalization, interpretability, and implementation.

### 9.1. Technical Challenges and Research Gaps

#### 9.1.1. Dataset Limitations and Reproducibility

A heavy emphasis in many studies has been laid on the fact, complemented by the presence of few high-quality and large-sized annotated datasets. Most bioacoustic studies lack full pipeline transparency in their results, as it is usually stated by Mutanu et al. [110]. They recognized qualities of reproducibility in the general consideration of studies, gaps in locomotion-related sounds, and inconsistent evaluation metrics being part of systemic issues. As recurring obstacles, Lavner and Pérez-Granados [74] describe low signal-to-noise ratios, class imbalance, and lack of global standardized datasets. Coutant et al. [111] conducted a scoping review of 52 bioacoustic studies across livestock species and identified common acoustic techniques and welfare indicators in this review. Inconsistencies in protocols and an increasing tendency toward ML-driven vocal analysis for automated welfare monitoring were also revealed in this report. This explains the need for standardization in poultry-focused bioacoustics.

#### 9.1.2. Cross-Domain Model Generalization

The question of whether models trained on one species or domain generalize to another is central to future applications. Van Merriënboer et al. [112] reviewed evaluation methods and showed how data variability and covariate shift affect degradation in generalization. Ghani et al. [35] and Gupta et al. [41] showed that transfer learning improves performance, but it still incurs a performance drop in unseen soundscapes or under polyphonic conditions. Swaminathan et al. [30] and Sarkar and Magimai-Doss [50] have shown that self-supervised models pretrained on human speech often outshine those trained from scratch but still require fine-tuning on animal-specific data.

There arises a gap, especially when transfer is considered from speech-pretrained models, such as wav2vec2 and Whisper, to the domain of poultry vocalizations. These models are trained on signals that resemble structured language, which include phonemic regularities and sentence-level dependencies. Calls from poultry are short, affective, continuous, or rhythmic, and lacking in segmental structures. In the absence of fine-tuning for the given task, these models may be unable to relate acoustic patterns to a meaningful biological interpretation.

#### 9.1.3. Domain Mismatch and Embedding Shift

One of the main challenges faced with transfer learning or indeed any self-supervised model for bioacoustics is domain mismatch, which produces embedding shifts where from one context of species/environment, feature representations learned would be misaligned in another environment. For example, models trained on chick or hen vocalizations usually fail to generalize to duck calls because calls are species-specific and can differ in harmonic structure, call duration, and frequency modulation. However, even within chickens, vocalizations vary across breed, age, and housing conditions, which confuses a classifier.

Swaminathan et al. [30] and Ghani et al. [35] observed that the fine-tuned wav2vec2 and PaSST models performed well and produced high accuracy within each specific dataset, yet embed drift occurred since they performed poorly or with reduced accuracy when tested on datasets of different species or recording setups. Ginovart-Panisello et al. [25] also reported failures in cross-breed generalization when training stress detection models on broiler vocalizations and applying them to laying hens. Such failures seemingly tell us that although modern deep learning models do possess very high capacity, latent features are not always biologically universal or invariant.

The existence of misalignment calls for an attempt at using domain adaptation, normalization of features across species, or unsupervised alignment of embedding spaces to bridge the gap between pretraining and deployment environments.

#### 9.1.4. Interpretability and Semantic Representation

Although many have succeeded in high classification rates, the number of works which deal with the interpretability of vocal signals is less. Neethirajan [68] and Cai et al. [72] both reached out to semantically decode chicken vocalizations with NLP-inspired models; however, the field has no broadly accepted benchmarks for semantic labeling or emotional annotations. Standard datasets, understandable architectures, and interdisciplinary interactions among acoustics, animal behavior, and machine learning are needed for future research efforts, according to Stowell [113].

While many advanced models, including CNNs, RNNs, and Transformers, manage to achieve high levels of classification accuracy, they are less interpretable, particularly so in realms requiring trust and transparency, such as animal welfare monitoring. The nature of the deep learning paradigm constitutes the so-called “black-box” problem, wherein the decision boundaries and internal logic remain opaque to end users such as veterinarians or farm operators. This increases the reluctance of such groups to deploy them in high-stakes environments where model explainability is itself a prerequisite for action and trust.

For instance, wav2vec2 and Whisper models achieve highly accurate classifications yet offer little insight into which vocal features or temporal patterns they rely on. Grad-CAM, SHAP, LIME, among others, are seldom used in bioacoustics; even when they are, the focus tends to be on spectrogram-level saliency as opposed to some biologically meaningful acoustic markers. A meaningful trade-off, hence, seems to exist between performance and interpretability, with the simpler models, probably SVMs or decision trees, giving less accuracy for more interpretability, whereas the more powerful deep models cast interpretability aside unless XAI features are explicitly incorporated within them.

### 9.2. Theoretical and Ethical Considerations

#### 9.2.1. Theoretical Foundations and Linguistic Analogs

Bolhuis et al. [114] reject claims of syntactic structure in bird vocalizations, stating that animal communication lacks true combinatorial semantics. Berthet et al. [101] support the importation of linguistic theories into animal communication (i.e., syntax, pragmatics) and argue that such models should respect certain ethological constraints. Jarvis [115] brought together many lines of research in vocal learning to suggest that animals might share features of language. However, the full accomplishment of vocal learning is rare and biologically constrained.

#### 9.2.2. Ethical Considerations

Currently, ethical studies are becoming very relevant in AI and animal research. Takeshita and Rzepka [116] identified numerous NLP datasets and models as embedding speciesism, thus warranting the need for the fair representation of nonhuman vocalizations in research and applications. Future studies should be concerned with multimodal systems and their use across a wider range of species. According to Zimmerman [65], Zimmerman and Koene [66], Manteuffel et al. [77], and Marino [117], there is a pressing need for further behavioral and emotional interpretations of poultry vocalizations. Morita et al. [52], Sainburg et al. [23], and Wang et al. [55], extended deep learning for modeling long-range dependencies, latent structures, and grammar-like patterns even in nonhuman species. Cross-species studies like Abzaliev et al. [49], Sethi et al. [96], and Bermant et al. [27] demonstrated that deep learning pipelines are highly adaptable but lack interpretability and standardization. The field is moving toward hybrid, explainable, and multi-species-aware models that better bridge computational power with ethological relevance.

From an ethical and practical standpoint, interpretability becomes even more crucial when AI systems are used to **make welfare-related decisions**. Uninterpretable models risk reinforcing biases, missing edge cases (e.g., rare distress calls), or overfitting to dataset noise without domain experts being able to audit the decisions. Therefore, future research must strike a balance between data-driven performance and transparent decision-making, possibly by integrating explainability modules directly into neural architectures or co-designing models with animal behaviorists.

### 9.3. Practical Gaps: Sensor Metrics, IoT Architecture, and Deployment Standards

Despite significant advances in algorithms, the real-world deployment of poultry acoustic AI systems faces practical challenges in sensor evaluation, wireless communication infrastructure, data fusion, and responsible technology design. One major limitation encountered in existing research is the absence of standardized metrics to define microphone and sensor robustness in the presence of a noisy farm environment. Benchmarking in the future should objectively report acoustic performance indicators such as sound-to-noise ratio (SNR), dB (A) ambient noise levels, and the attenuation profile in the frequency bands of interest. Potential techniques for noise cancellation can be explored and applied, for example, through spectral subtraction, Wiener filtering, and neural-based speech enhancement, all promising to improve the performance of the system under heavy noise conditions [118]. The on-farm deployment is also largely dependent on the proper selection of the wireless protocols: Technologies have different trade-offs between cost, latency, and energy efficiency. Recently, LoRaWAN has gained attention for its extremely low power consumption and maximum range of 5–15 km, NB-IoT is available as a carrier-integrated medium-bandwidth solution, and Zigbee works in the short range with mesh networking capabilities. For instance, Zigbee is suitable for local mesh needs in densely populated poultry houses, whereas LoRaWAN would provide long-range coverage for widely spaced farms. Those compromises directly affect the system’s acoustic scale and interoperability and should, therefore, be explicitly considered when planning the infrastructure [119].

Acoustic surveillance systems should comply with both data privacy and sustainability objectives. In the European Union, any system collecting or storing identifiable vocalizations must comply with the General Data Protection Regulation (GDPR) [120]. In parallel, considerations about the rampant deployment of embedded sensors being an electronic waste problem have also emerged. Research now emphasizes sustainable smart farming practices, such as modular sensor designs, recyclable components, and low-power architecture as a means to reduce e-waste and ensure long-term viability [121].

Rare vocalization types—created, for example, to signal the onset of a disease or acute distress—often have limited labeled data. Few-shot learning frameworks, with Prototypical Networks (ProtoNets) being a classical example, provide a way to classify these infrequent events reliably from only very few examples [122]. In order to achieve deployment transparency, XAI solutions can be used. For instance, Grad-CAM or LIME visualization techniques [123] can highlight the regions of spectrograms that influence CNN model decision-making, thus helping to boost model trust and, in turn, farmer acceptance. Adoption ultimately hinges on the alignment of the system with a farmer’s workflow and usability expectations. Interface formats (e.g., SMS alerts vs. dashboard visualizations), economic modeling (e.g., $50/sensor vs. 10% mortality reduction), and participatory design strategies (e.g., focus groups, usability trials) must be employed for development. Training may be given through applications such as DeepSqueak [124] that will allow farmers and technicians to actively engage in annotation, validation, and deployment, cultivating long-lasting adoption and trust toward the technology.

## 10. Conclusions

This systematic review unveils a rapidly transforming landscape where artificial intelligence fundamentally redefines our understanding of animal communication and welfare assessment through poultry vocalizations. Our comprehensive analysis of over 120 studies reveals a decisive paradigm shift from traditional hand-crafted acoustic features toward sophisticated self-supervised learning architectures, with models like wav2vec2 and SincNet demonstrating unprecedented capabilities in decoding the complex emotional and physiological states embedded within avian vocalizations. The convergence of bioacoustics and machine learning has reached a critical inflection point, where theoretical advances in deep learning architectures now demand practical translation into robust, deployable farm-level systems. However, our investigation exposes fundamental challenges that threaten to impede widespread adoption: the persistent opacity of black-box models undermines stakeholder trust, cross-species generalization remains elusive despite sophisticated transfer learning approaches, and the absence of standardized evaluation frameworks creates a fragmented research ecosystem that hinders reproducible science.

The interpretability crisis emerges as perhaps the most pressing concern for real-world deployment. While achieving impressive classification accuracies exceeding 95% in controlled settings, current deep learning models operate as impenetrable decision-making systems, providing little insight into which acoustic signatures drive welfare assessments. This opacity becomes particularly problematic when veterinarians and farm operators must act upon AI-generated alerts, demanding explainable artificial intelligence solutions that balance performance with transparency. Domain adaptation challenges reveal the brittleness of current approaches when deployed across diverse poultry breeds, housing conditions, and environmental contexts. Models trained on broiler vocalizations frequently fail when applied to laying hens, while embedding drift causes performance degradation when acoustic environments shift from laboratory to commercial farm settings. This limitation threatens the scalability of AI-driven welfare monitoring systems across the heterogeneous landscape of global poultry production.

The integration of edge computing and TinyML frameworks presents both unprecedented opportunities and technical constraints for continuous welfare monitoring. While enabling real-time inference directly on farm hardware, these resource-constrained deployments demand architectural innovations that maintain model performance while operating within strict power and computational budgets. Future trajectories must prioritize the development of interpretable, domain-adaptive models that seamlessly integrate multimodal sensor data while maintaining ethical standards for animal welfare assessment. The establishment of standardized benchmarking protocols, cross-species evaluation frameworks, and transparent dataset sharing initiatives will determine whether this promising field evolves into a transformative technology for precision livestock farming or remains confined to academic research.

The stakes extend beyond technological advancement—they encompass our fundamental responsibility to ensure that AI systems designed to safeguard animal welfare operate with the transparency, reliability, and ethical grounding that both animals and their human caretakers deserve.

## Figures and Tables

**Table 2:** Performance of classical machine learning models in animal vocalization classification.

Authors	Model(s)	Reported Accuracy
Pereira et al. [8]	Random Forest	85.61%
Tao et al. [26]	SVM, RF, k-NN	94.16%
Du et al. [31]	SVM	Sensitivity = 95.1%
Bhandekar et al. [36]	SVM	95.66%
Ginovart-Panisello et al. [37]	Gaussian Naive Bayes	F1-score = 80%

**Table 5:** Top 5 general poultry vocalization classifiers.

Model (Author)	Reported Performance	Noise Robustness	Species/Use Case	Inference Efficiency	Reference
SincNet (Bravo Sanchez et al.)	>65% accuracy	High	Songbirds	Extremely efficient (low params)	[5]
Light-VGG11 (Mao et al.)	95% accuracy	High (on-farm)	Chicken	Good; 92.78% parameter reduction	[19]
Conv1D + Burn Layer (Hassan et al.)	98.55% accuracy	High	Chicken (distress detection)	Lightweight; optimized for edge	[32]
MFF-ScSEnet (Hu et al.)	>96% accuracy	Moderate–High	Birdsong	Medium; attention module	[34]
CNN-LMU (Gupta et al.)	Best in benchmark	Moderate	Songbirds	Compact recurrent unit	[41]

**Table 7:** Comparison of common microphone and acoustic sensor types used in on-farm poultry acoustic monitoring, highlighting trade-offs in signal quality, power, and deployment suitability.

Sensor Type	Example Devices	Sampling Rate	SNR	Power Consumption	Form Factor	Cost (Estimate)	Remarks
Piezoelectric	ChickenSense (custom) [86]	16–44.1 kHz	Moderate	Very Low	Contact-mount	Low (<$5)	Good for contact-based feeding detection
MEMS Microphone	ReSpeaker USB Mic Array	48 kHz	63–72 dB	Low	Beamforming array	Moderate ($25–40)	Enables directional detection and active noise cancellation
Electret Condenser	Analog mic modules	8–16 kHz	Low–Mid	Moderate	Analog circuit	Very Low (~$2)	Noisy, often used in low-cost setups
MEMS + DSP (digital)	Syntiant NDP101 + mic front-end	16–32 kHz	High	Ultra Low (<1 mW)	Edge-ML enabled	Moderate–High ($40+)	Optimized for TinyML and keyword spotting

**Table 8:** IoT protocols for poultry acoustic + sensor monitoring.

Protocol	Range	Bandwidth	Power Efficiency	Cost	Best For	Limitations
LoRaWAN	5–15 km (rural)	Low (0.3–50 kbps)	Excellent	Low to Mod	Long-range farm monitoring	Latency, not for high-frequency data
Zigbee	~10–100 m	Medium (250 kbps)	Good	Low	Local mesh in dense poultry houses	Needs mesh routers, limited range
NB-IoT	1–10 km (urban)	Low–Med (26–127 kbps)	Excellent	Carrier tied	Cellular farms w/good coverage	Carrier dependency, SIM/data needed
Wi-Fi	~100 m	High (Mbps)	Poor	Moderate	Real-time dashboards and video	Power-hungry, not suitable for edge AI
BLE 5.0	~100–400 m	Low (~2 Mbps)	Excellent	Low	Low-power sensor pairing	Short range, not ideal for big farms

## Data Availability

No new data were created or analyzed in this study. Data sharing is not applicable to this article.
